# Porcine Reproductive and Respiratory Syndrome Virus nsp5 Induces Incomplete Autophagy by Impairing the Interaction of STX17 and SNAP29

**DOI:** 10.1128/spectrum.04386-22

**Published:** 2023-02-23

**Authors:** Yanrong Zhou, Yang Li, Ran Tao, Jia Li, Liurong Fang, Shaobo Xiao

**Affiliations:** a State Key Laboratory of Agricultural Microbiology, College of Veterinary Medicine, Huazhong Agricultural University, Wuhan, China; b Key Laboratory of Preventive Veterinary Medicine in Hubei Province, Cooperative Innovation Center for Sustainable Pig Production, Wuhan, China; Changchun Veterinary Research Institute

**Keywords:** porcine reproductive and respiratory syndrome virus, nonstructural protein 5, autophagy, syntaxin 17, synaptosomal-associated protein 29

## Abstract

Porcine reproductive and respiratory syndrome virus (PRRSV) is an economically important pathogen that has devastated the worldwide swine industry for over 30 years. Autophagy is an evolutionarily conserved intracellular lysosomal degradation pathway, and previous studies have documented that PRRSV infection prompts autophagosome accumulation. However, whether PRRSV induces complete or incomplete autophagy remains controversial. Here, we demonstrated that overexpression of PRRSV nonstructural protein 5 (nsp5) induced the accumulation of autophagosomes, and a similar scenario was observed in PRRSV-infected cells. Moreover, both PRRSV infection and nsp5 overexpression activated incomplete autophagy, as evidenced by the blockage of autophagosome-lysosome fusion. Mechanistically, nsp5 overexpression, as well as PRRSV infection, inhibited the interaction of syntaxin 17 (STX17) with synaptosomal-associated protein 29 (SNAP29), two SNARE proteins that mediate autophagosome fusion with lysosomes, to impair the formation of autolysosomes. We further confirmed that nsp5 interacted with STX17, rather than SANP29, and the interacting domains of STX17 were the N-terminal motif and SNARE motif. Taken together, the findings of our study suggest a mechanism by which PRRSV induces incomplete autophagy by blocking autophagosome degradation and provide insights into the development of new therapeutics to combat PRRSV infection.

**IMPORTANCE** A substantial number of viruses have been demonstrated to utilize or hijack autophagy to benefit their replication. In the case of porcine reproductive and respiratory syndrome virus (PRRSV), previous studies have demonstrated the proviral effects of autophagy on PRRSV proliferation. Thus, an investigation of the mechanism by which PRRSV regulates the autophagy processes can provide new insight into viral pathogenesis. Autophagic flux is a dynamic process that consists of autophagosome formation and subsequent lysosomal degradation. However, the exact effect of PRRSV infection on the autophagic flux remains disputed. In this study, we demonstrated that PRRSV infection, as well as PRRSV nsp5 overexpression, inhibited the interaction of STX17 with SNAP29 to impair the fusion of autophagosomes with lysosomes, thereby blocking autophagic flux. This information will help us to understand PRRSV-host interactions and unravel new targets for PRRS prevention and control.

## INTRODUCTION

Autophagy is a fundamental intracellular recycling process that widely exists in eukaryotic cells (1). It is initiated via phagophores, which expand to become bilayer structures called autophagosomes, which subsequently fuse with lysosomes to form degradative autolysosomes. Ultimately, engulfed cytoplasmic constituents, such as proteins, lipids, nucleic acids, and damaged or unwanted organelles, within autolysosomes are degraded to facilitate recycling of amino acids, fatty acids, nucleotides, and energy (2, 3). Complete autophagic flux is critical in combating external stress stimuli, such as starvation, and for the maintenance of cellular homeostasis. However, an increasing number of studies have demonstrated that some kinds of stimuli cause cells to undergo mutilated-autophagic responses, resulting in impaired autophagosome-lysosome fusion and the subsequent blockage of autophagic flux, namely, incomplete autophagy (4).

In mammalian cells, soluble *N*-ethylmaleimide-sensitive factor attachment protein receptor (SNARE) proteins are established molecular drivers of membrane fusion, including the fusion of autophagosomes and lysosomes. SNARE proteins are classified into four groups: Qa, Qb, Qc, and R-SNAREs (5, 6). Generally, one SNARE protein in each group constitutes the SNARE complex, which mediates membrane fusion. In terms of the autophagy process, Qa-SNARE STX17 is recruited to the outer membrane of a mature autophagosome to interact with cytosolic Qbc-SNARE SNAP29 and lysosomal R-SNARE VAMP8 to form the SNARE complex STX17-SNAP29-VAMP8, which directly mediates autophagosome-lysosome physical fusion, also called autophagosome maturation (7, 8). The efficiency of SNARE-dependent membrane fusion is determined to be modulated by diverse intrinsic or extrinsic factors, including host proteins, such as homotypic fusion and vacuole protein-sorting complex, autophagy-related 14, and Rab GTPase (9–11); the modification of SNAREs (7, 12); drugs (13, 14); and virus infections (15, 16).

Porcine reproductive and respiratory syndrome (PRRS) has caused great economic loss to the global pig industry since its discovery in the late 1980s (17). The etiological agent PRRS virus (PRRSV) is a positive-sense single-stranded RNA virus belonging to the *Nidovirales* order of the *Arteriviridae* family (18). The PRRSV genome is approximately 15 kb in length and contains at least 10 open reading frames (ORF1a, ORF1b, ORF2a, ORF2b, ORF3 to ORF7, and ORF5a). ORF1a and ORF1b encode the pp1a and pp1ab polyproteins, respectively, which can be cleaved into at least 14 mature nonstructural proteins (nsps), including nsp1α, nsp1β, nsp2 to nsp6, nsp7α, nsp7β, and nsp8 to nsp12 (19, 20). Previous studies suggested that PRRSV infection can hijack autophagy to benefit its proliferation, and PRRSV infection induces autophagosome accumulation (21–23). The accumulation of autophagosomes occurs when autophagosome formation is increased or autophagosome-lysosome fusion is blocked (24). Presently, whether autophagosomes fuse with lysosomes in PRRSV-infected cells to induce complete autophagy (23, 25) or autophagosomes do not fuse with lysosomes, which induces incomplete autophagy (26, 27), remains disputed. To date, three PRRSV-encoded nonstructural proteins, nsp2, nsp3, and nsp5, have been reported to modulate the levels of 1A/1B-light chain 3 II (LC3-II)/LC3-I and p62 (the classical autophagy markers). Nsp2 has been demonstrated to colocalize with LC3 and induce autophagy in a manner dependent on aggresome formation (26, 28), and nsp3 appears to induce autophagosome formation through its cytoplasmic domain (27). However, the exact mechanisms involved in nsp5-mediated autophagy regulation have not been systematically investigated.

In this study, we identified that PRRSV infection induces incomplete autophagy, and PRRSV nsp5 is responsible for autophagosome accumulation by inhibiting autophagosome-lysosome fusion. Of significant interest, nsp5 reduced SNARE complex (STX17-SNAP29-VAMP8) formation by interacting with the N-terminal and SNARE domains of STX17, thereby blocking autophagic flux.

## RESULTS

### PRRSV nsp5 induces autophagosome accumulation.

**FIG 1:**
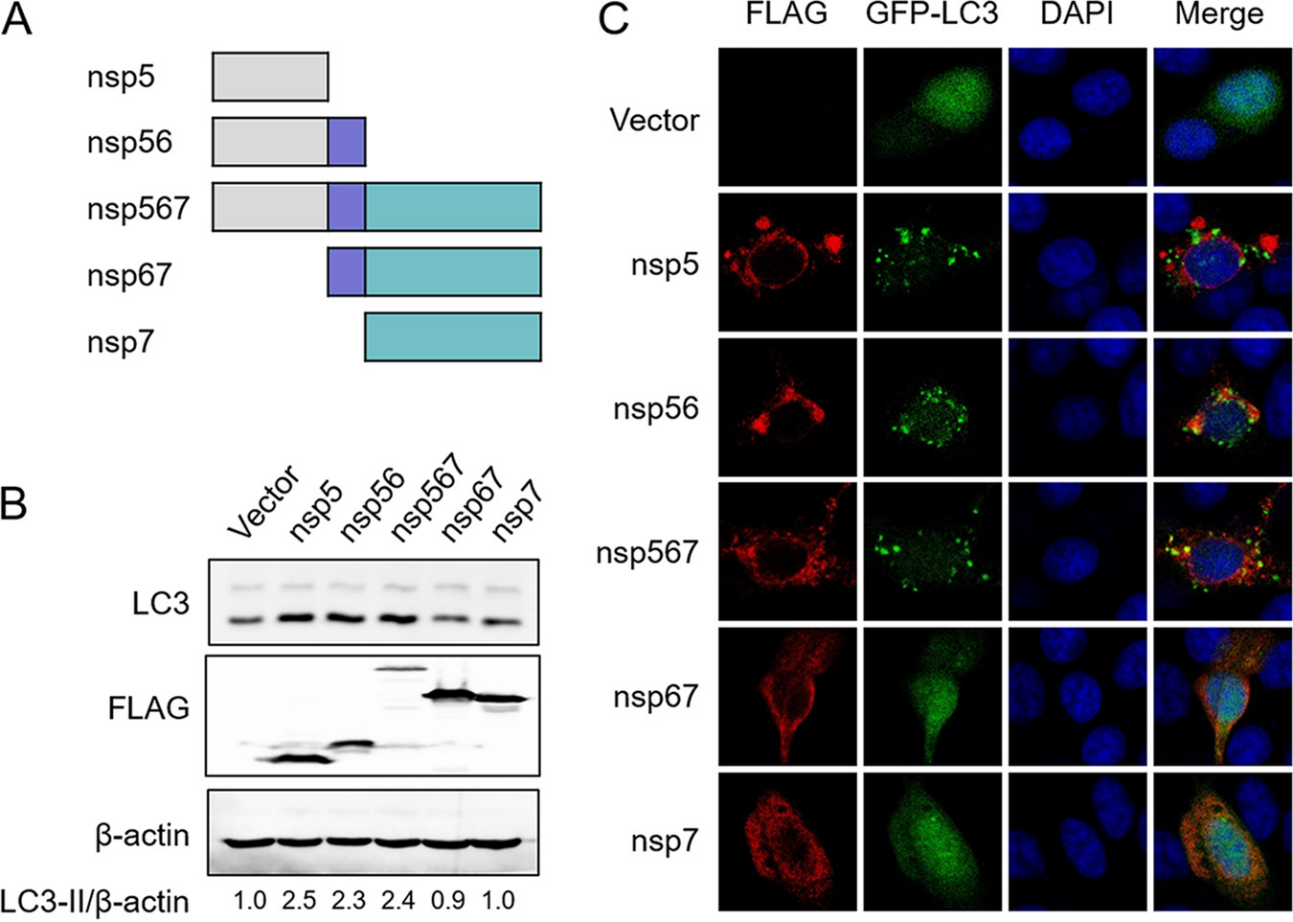
Overexpression of PRRSV nsp5 triggers the accumulation of autophagosomes. (A) Schematic representation of PRRSV nsp5, nsp56, nsp567, nsp67, and nsp7. The sequences were cloned into pCAGGS vectors to construct expression plasmids. (B) HEK-293T cells were transfected with FLAG-tagged plasmids encoding nsp5, nsp56, nsp567, nsp67, or nsp7. After 30 h, the cells were harvested for Western blot analysis to detect the expression levels of LC3. Relative LC3-II protein levels were quantified by ImageJ. (C) HEK-293T cells were cotransfected with plasmids encoding GFP-LC3 and FLAG-tagged nsp5, nsp56, nsp567, nsp67, or nsp7. After 30 h, indirect immunofluorescence assay was conducted to detect the formation of GFP-LC3 puncta. Nuclei were stained with DAPI (blue).

A previous study showed that coronavirus nsp6, equivalent to the PRRSV nsp5-7 precursor protein, induces autophagosome accumulation (29). Another study reported that PRRSV nsp5 may be involved in the regulation of autophagy (27). To clarify whether PRRSV nsp5 alone can induce autophagosome accumulation, we constructed expression plasmids encoding nsp5, nsp56, nsp567, nsp67, and nsp7 (Fig. 1A) and investigated the effects of the expressed proteins on the localization and levels of LC3 using immunoblotting assays and immunofluorescence analysis, respectively. As shown in Fig. 1B, all constructs expressing nsp5, including nsp5, nsp56, and nsp567, upregulated the levels of LC3-II, a hallmark of autophagosome formation, but those expressing nsp67 and nsp7 did not, suggesting that nsp5 is critical for inducing autophagy. In line with this observation, we found that overexpression of nsp5, nsp56, or nsp567 resulted in a remarkable increase in green fluorescent protein (GFP)-LC3 puncta formation (Fig. 1C). Together, these results indicated that nsp5 induced autophagosome accumulation.

### PRRSV nsp5 is an ER-localized protein but hardly induces ER stress.

**FIG 2:**
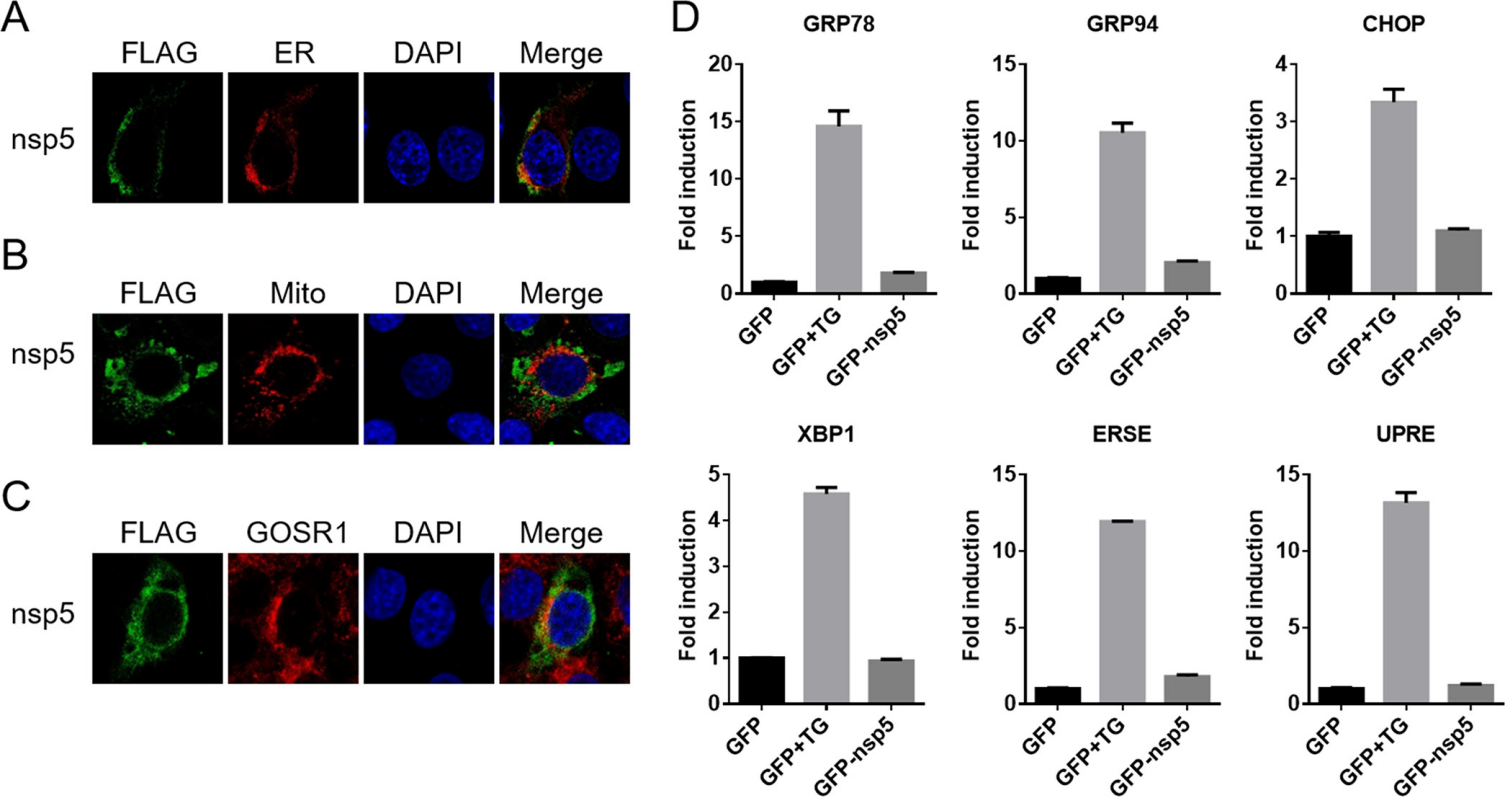
Nsp5 localizes to the endoplasmic reticulum (ER) but induces no ER stress. (A and B) MARC-145 cells were cotransfected with FLAG-nsp5 and ER marker pDsRed-ER (A) or mitochondria marker DsRed2-Mito (B). Thirty hours later, indirect immunofluorescence assay was performed, with nsp5 detected by anti-FLAG antibody. Nuclei were stained with DAPI (blue). (C) MARC-145 cells were transfected with FLAG-nsp5. After 30 h, an indirect immunofluorescence assay was conducted, with nsp5 detected by anti-FLAG antibody and Golgi labeled by anti-GOSR1 antibody. (D) HEK-293T cells were transfected with plasmids expressing GFP or GFP-tagged nsp5 together with reporter plasmid (GRP78-*luc*, GRP94-*luc*, CHOP-*luc*, XBP-1-*luc*, UPRE-*luc*, or ERSE-*luc*) and pRL-TK plasmid. Cells treated with TG (5 μM) for 4 h were used as positive controls. Thirty hours after transfection, the cells were harvested for luciferase reporter assays.

The dysfunction of membrane-bound organelles, such as the endoplasmic reticulum (ER), mitochondria, and Golgi, has been reported to contribute to autophagy activity (30–32). Given that PRRSV nsp5 is a hydrophobic transmembrane protein, we first detected its subcellular localization. MARC-145 cells were transfected with plasmids pCAGGS-FLAG-nsp5 and/or pDsRed-ER (an ER marker) and pDsRed2-Mito (a mitochondria marker). Endogenous Golgi SNAP receptor complex member 1 (GOSR1), a Golgi marker, was stained with a specific antibody. As shown in Fig. 2A, nsp5 mainly colocalized with the ER and was largely separated from the mitochondria (Fig. 2B) and Golgi (Fig. 2C), indicating that nsp5 is a potential ER transmembrane protein.

ER stress is a well-known trigger for autophagy in mammalian cells (33). To deal with ER stress, the unfolded protein response (UPR) is initiated to eliminate misfolded or unfolded proteins through three pathways: the protein kinase R-like ER kinase (PERK), activating transcription factor 6 (ATF6), and inositol-requiring enzyme 1 (IRE1) pathways (34). Previous studies have indicated that PRRSV infection induces ER stress and UPR (35), and thus we speculated that PRRSV nsp5 is likely to manipulate autophagy by inducing ER stress and UPR. To test the validity of this speculation, we analyzed the effect of nsp5 on the promoter activities of c/EBP homologous protein (CHOP; the hallmark of both the PERK and ATF6 pathways), X-box-binding protein 1 (XBP1; the hallmark of the IRE1 pathway), glucose-regulated protein 78 (GRP78) and GRP94 (two ATF6-responsive genes), and ER stress elements (ERSE) and UPR elements (UPRE). Thapsigargin (TG) treatment was set as the positive control. As shown in Fig. 2D, contrary to the effect of TG, no obvious increase was detected in the activity of CHOP, XBP1, GRP78, GRP94, ERSE, or UPRE, suggesting that nsp5 did not induce UPR or ER stress. We interpreted the findings to mean that, although PRRSV nsp5 is an ER-localized protein, nsp5-mediated autophagosome accumulation may be induced in an ER stress-independent manner.

### PRRSV nsp5 blocks autophagosome-lysosome fusion.

**FIG 3:**
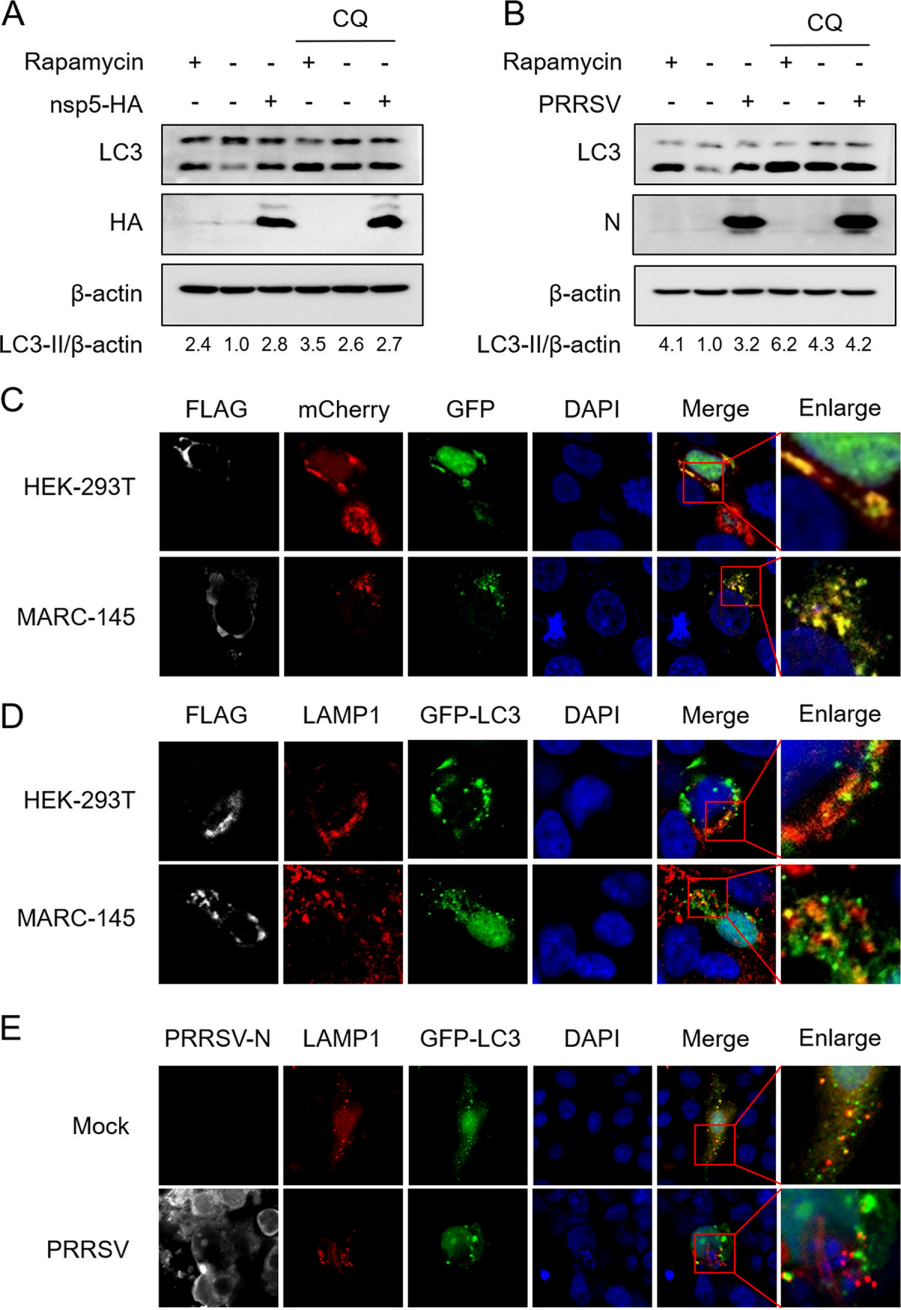
PRRSV nsp5 induces incomplete autophagy. (A and B) HEK-293T cells were transfected with plasmids expressing HA-tagged nsp5 or empty vector (A), and MARC-145 cells were infected with PRRSV (multiplicity of infection [MOI], 0.5) (B). After 24 h, cells were treated or mock treated with CQ (20 μM) for another 12 h. Rapamycin treatment (10 μM) for 6 h served as the positive inducer of complete autophagy. The cells were lysed for Western blot analysis. (C) HEK-293T or MARC-145 cells were cotransfected with pmCherry-EGFP-LC3 and pCAGGS-FLAG-nsp5. Thirty hours later, cells were harvested for fluorescence analysis. (D) HEK-293T or MARC-145 cells were cotransfected with pLAMP1-mCherry, pEGFP-LC3 and pCAGGS-FLAG-nsp5. Thirty hours later, cells were harvested for fluorescence analysis. (E) MARC-145 cells were cotransfected with pEGFP-LC3 and pLAMP1-mCherry. After 12 h, cells were infected with PRRSV (MOI, 0.5). Thirty-six hours later, cells were processed for immunofluorescence assay. PRRSV-N monoclonal antibody was used to detect PRRSV-infected cells (white). Nuclei were stained with DAPI (blue).

Autophagosome accumulation happens when autophagic flux is induced or autophagosome degradation is blocked (36). To explore the mechanism of autophagosome accumulation induction by nsp5, HEK-293T cells were transfected with hemagglutinin (HA)-tagged plasmids expressing nsp5, followed by treatment with chloroquine (CQ; 20 μM), which induces autophagosome accumulation by impairing autophagosome degradation. Rapamycin (10 μM) was used as a positive inducer of complete autophagy. The results showed that, compared with the untreated group, the levels of LC3-II were increased by nsp5 overexpression in the absence of CQ but remained unchanged after nsp5 overexpression in the presence of CQ (Fig. 3A), suggesting that nsp5 induces autophagosome accumulation by inhibiting autophagosome degradation.

To test whether PRRSV infection also blocks the degradation of autophagosomes, MARC-145 cells were infected with PRRSV and then treated with CQ (20 μM) at 24 h postinfection for another 12 h. Consistent with the effects of nsp5 overexpression, PRRSV infection did not lead to a further accumulation of LC3-II when autophagic degradation was blocked by CQ (Fig. 3B), suggesting that autophagosome accumulation during PRRSV infection occurs due to the blockage of autophagosome-lysosome fusion.

The GFP signal tends to be quenched in the acidic and proteolytic lysosome lumen, whereas mCherry is more stable (37). Therefore, a tandem mCherry-enhanced GFP (EGFP)-LC3 fluorescence protein is commonly applied to monitor autophagic flux, with red LC3 puncta (positive for mCherry signal and negative for GFP signal) indicating the formation of autolysosomes and yellow LC3 puncta (positive for both mCherry and GFP signals) representing an impairment in autophagosome-lysosome fusion (38). Here, HEK-293T cells and MARC-145 cells were cotransfected with plasmids expressing FLAG-tagged nsp5 and tandem mCherry-EGFP-LC3 fluorescent protein. As shown in Fig. 3C, yellow LC3 puncta accumulated in both HEK-293T cells expressing nsp5 and MARC-145 cells expressing nsp5, suggesting that nsp5 blocked the fusion of autophagosomes with lysosomes. To strengthen this conclusion, we further investigated whether nsp5 affected the colocalization of GFP-LC3 puncta with LAMP1, a biological marker of lysosomes (24). HEK-293T cells and MARC-145 cells were cotransfected with plasmids expressing FLAG-tagged nsp5, GFP-LC3, and pLAMP1-mCherry. As shown in Fig. 3D, GFP-LC3 puncta were largely negative for LAMP1 in both HEK-293T cells and MARC-145 cells that expressed nsp5, indicating that nsp5 overexpression inhibited the fusion of autophagosomes with lysosomes. Similarly, we found no obvious colocalization of GFP-LC3 puncta and LAMP1 in PRRSV-infected MARC-145 cells (Fig. 3E), demonstrating that autophagosomes failed to fuse with lysosomes in the context of PRRSV infection, which concurs with the observation in nsp5-expressing cells. Notably, nsp5 could also block the fusion of autophagosomes with lysosomes induced by nutrient deprivation or rapamycin (see Fig. S1 in the supplemental material).

### PRRSV nsp5 attenuates the interaction between STX17 and SNAP29.

**FIG 4:**
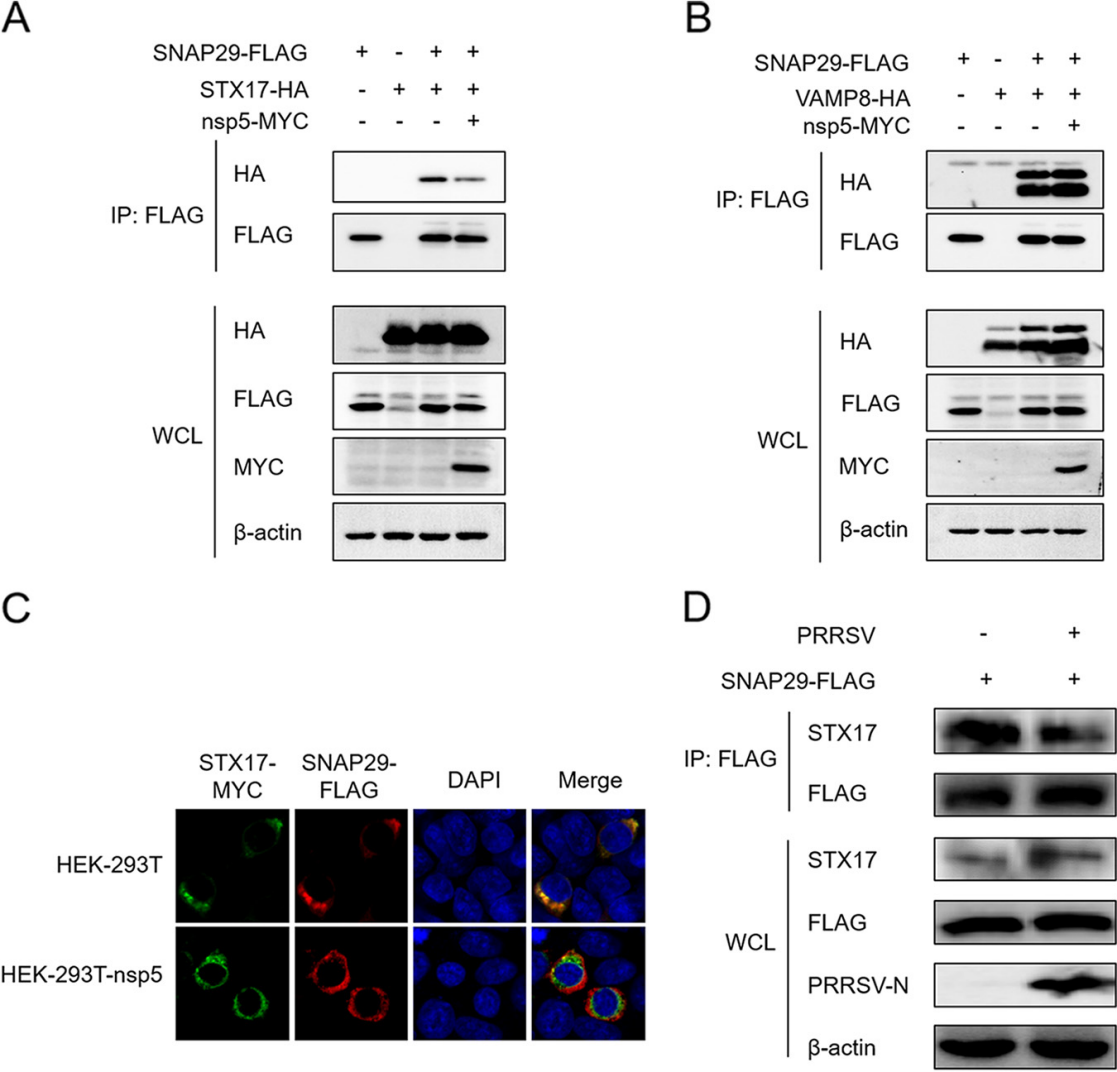
PRRSV nsp5 inhibits the interaction between STX17 and SNAP29. (A and B) HEK-293T cells were cotransfected with pCAGGS-SNAP29-FLAG, pCAGGS-nsp5-MYC, and pCAGGS-STX17-HA (A) or pCAGGS-VAMP8-HA (B). Thirty hours later, the cells were lysed for co-IP assays. FLAG monoclonal antibody was used to precipitate SNAP29 protein. Protein levels were measured by Western blot analysis. (C) HEK-293T cells or HEK-293T cells stably expressing nsp5 (HEK-293T-nsp5) were treated with 1 μg/mL doxycycline for 12 h. Then, cells were cotransfected with pCAGGS-STX17-MYC and pCAGGS-SNAP29-FLAG. Twenty-four hours later, cells were harvested for immunofluorescence assay. Nuclei were stained with DAPI (blue). (D) MARC-145 cells were transfected with pCAGGS-SNAP29-FLAG. After 24 h, cells were infected with PRRSV (MOI, 0.5). Thirty-six hours later, the cells were lysed for co-IP assays. FLAG monoclonal antibody was used to precipitate the SNAP29 protein. Protein levels were measured by Western blot analysis.

Fusion of autophagosomes with lysosomes in mammals is predominantly mediated by the autophagic SNARE complex (STX17-SNAP29-VAMP8) (39). We next examined the effect of nsp5 on the formation of STX17-SNAP29-VAMP8. HEK-293T cells were cotransfected with plasmids expressing nsp5, STX17, and SNAP29. As shown in Fig. 4A, in a FLAG-Trap assay, the level of STX17 coprecipitated with FLAG-SNAP29 was substantially reduced by nsp5 overexpression. However, the interaction between SNAP29 and VAMP8 remained unchanged in cells expressing nsp5 (Fig. 4B). The immunofluorescence assay also showed that SNAP29 colocalization with STX17 was dramatically reduced in HEK-293T cells stably expressing nsp5 (Fig. 4C). These data suggest that nsp5 disrupts the interaction between STX17 and SNAP29, thereby disrupting the assembly of the STX17-SNAP29-VAMP8 complex to impair autophagosome-lysosome fusion. To further investigate whether PRRSV infection also affects STX17 interaction with SNAP29, MARC-145 cells were transfected with FLAG-SNAP29 and then infected with PRRSV. As shown in Fig. 4D, the level of endogenous STX17 precipitated with FLAG-SNAP29 was remarkably reduced in PRRSV-infected cells, suggesting that PRRSV infection blocked the formation of functional STX17-SNAP29-VAMP8 complexes.

### PRRSV nsp5 interacts with the N-terminal and SNARE domains of STX17.

**FIG 5:**
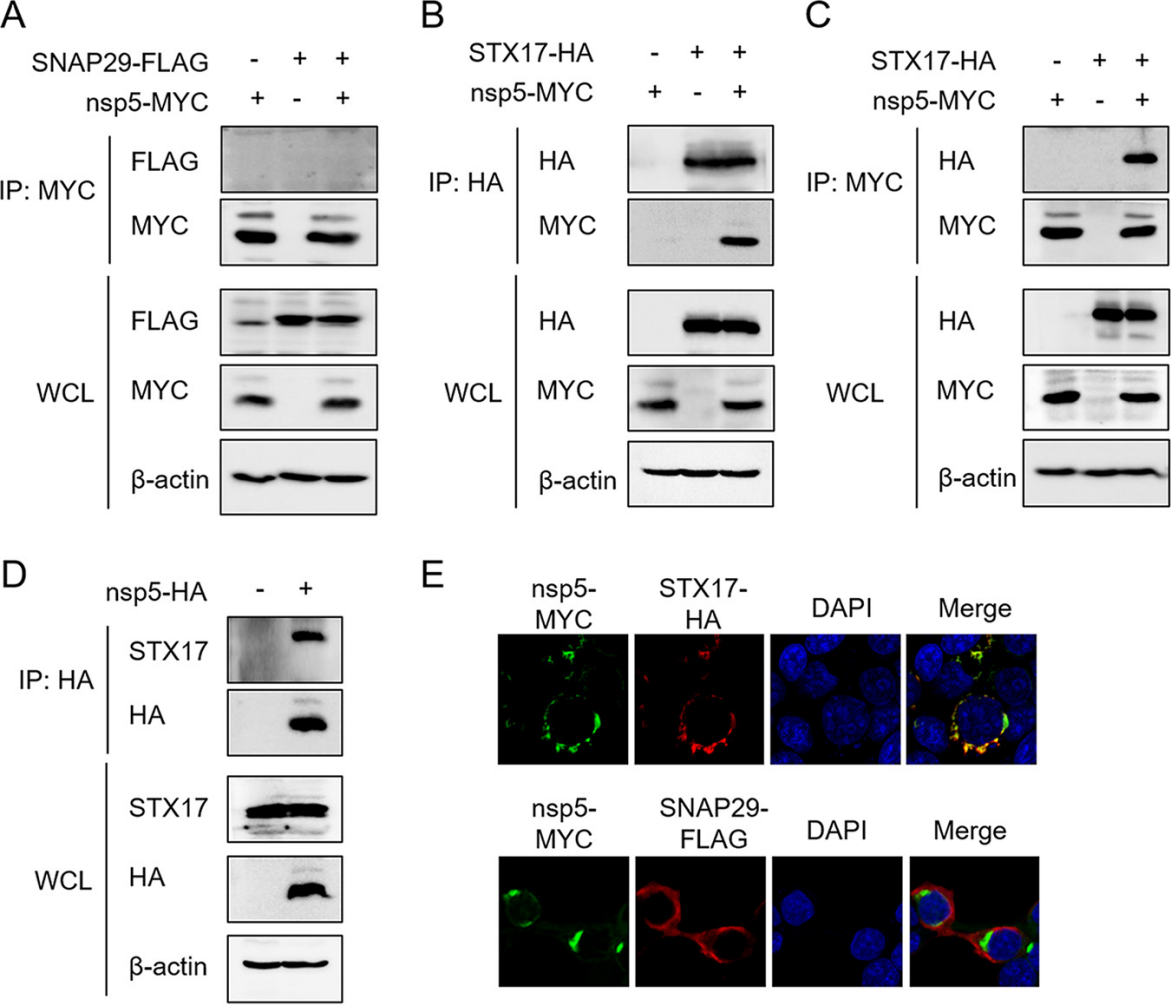
PRRSV nsp5 interacts with STX17. (A) HEK-293T cells were cotransfected with pCAGGS-SNAP29-FLAG and pCAGGS-nsp5-MYC. The cells were harvested for co-IP assay 30 h later. MYC monoclonal antibody was used to precipitate nsp5. Protein levels were measured by Western blot assay. (B and C) HEK-293T cells were cotransfected with pCAGGS-STX17-HA and pCAGGS-nsp5-MYC. The cells were harvested for co-IP assay 30 h later. HA monoclonal antibody (B) or MYC monoclonal antibody (C) was used to precipitate STX17 (B) or nsp5 (C). Protein levels were measured by Western blot assay. (D) HEK-293T cells were transfected with pCAGGS-nsp5-HA. Thirty hours later, the cells were lysed for co-IP assay and an HA monoclonal antibody was used to precipitate nsp5. Protein levels were measured by Western blot assay. (E) HEK-293T cells were cotransfected with pCAGGS-nsp5-MYC and pCAGGS-STX17-HA or pCAGGS-SNAP29-FLAG. Thirty hours later, the cells were harvested for immunofluorescence assay. Nuclei were stained with DAPI (blue).

We next determined whether PRRSV nsp5 disrupts the interaction between STX17 and SNAP29 by interacting with STX17 and/or SNAP29. HEK-293T cells were cotransfected with plasmids expressing MYC-tagged nsp5 and FLAG-tagged SANP29 or HA-tagged STX17. In the MYC-Trap assay, nsp5 showed no interaction with SNAP29 (Fig. 5A), while STX17 was strongly precipitated (Fig. 5B). Consistent with this, interaction between nsp5 and STX17 was also observed in a HA-Trap assay (Fig. 5C). We also found that nsp5 interacted with endogenous STX17 in HEK-293T cells transfected with HA-tagged nsp5 (Fig. 5D). Additionally, the obvious colocalization of nsp5 and STX17, but not nsp5 and SNAP29, was observed in the indirect immunofluorescence assay (Fig. 5E). These results indicated that nsp5 interacts with STX17.

**FIG 6:**
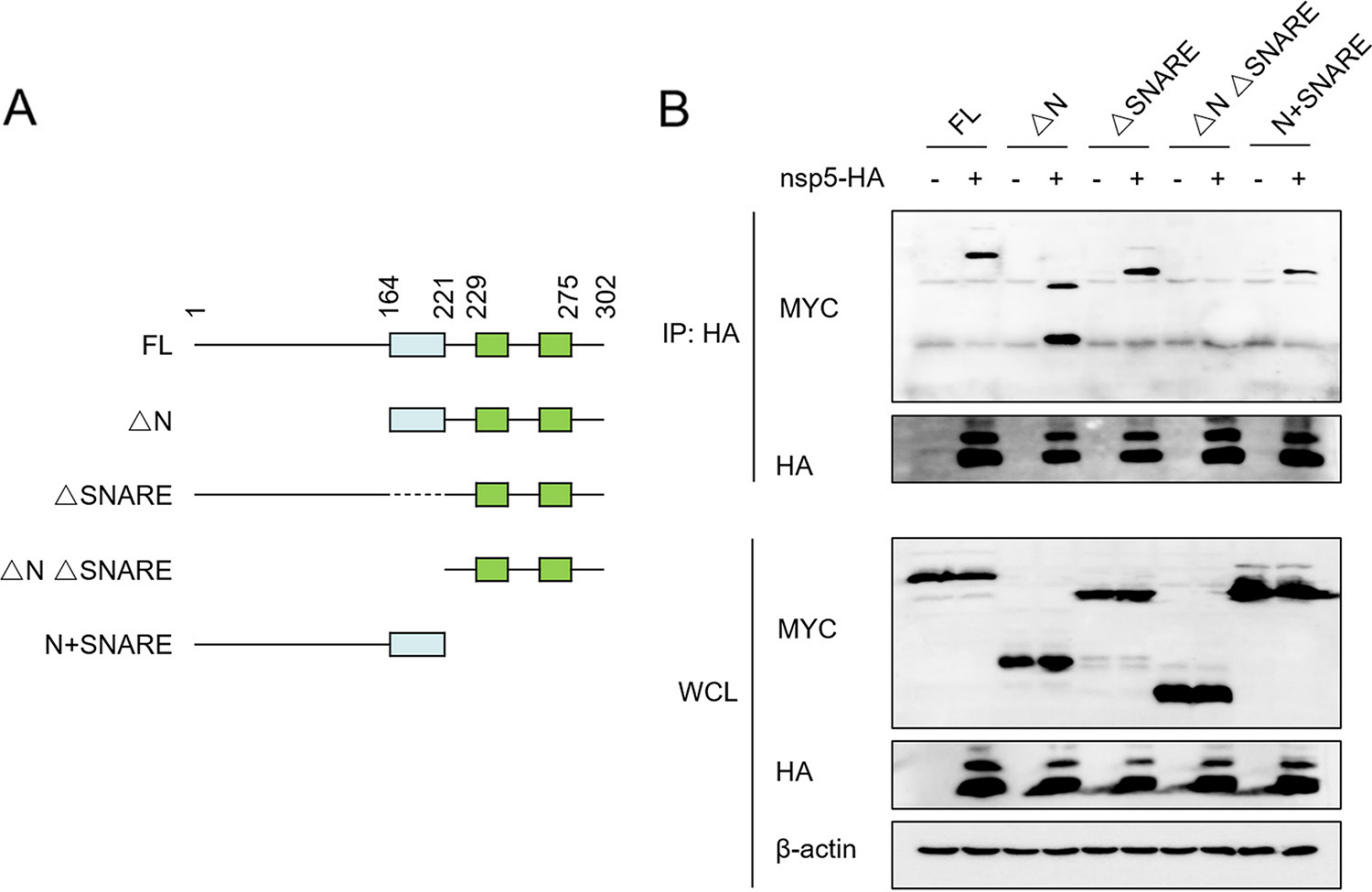
PRRSV nsp5 interacts with the N-terminal domain and SNARE domain of STX17. (A) Truncated mutants (ΔN, ΔSNARE, ΔNΔSNARE, and N+SNARE) of STX17 were inserted into pCAGGS-MYC as indicated. (B) HEK-293T cells were cotransfected with plasmids expressing nsp5-HA and STX17-MYC or its truncated mutants. After 30 h, the cells were lysed for co-IP assays using an HA monoclonal antibody. Protein levels were measured by Western blot assay.

To further identify the domain(s) of STX17 that interacts with nsp5, we constructed truncated STX17 mutants, including ΔN (deletion of amino acids [aa] 1 to 164), ΔSNARE (deletion of aa 165 to 221), ΔNΔSNARE (deletion of aa 1 to 221), and N+SNARE (aa 1 to 221) (Fig. 6A). These mutants and the nsp5 expression plasmids were cotransfected into HEK-293T cells for coimmunoprecipitation (co-IP) experiments. The results showed that nsp5 interacted with STX17 ΔN, ΔSNARE, and N+SNARE truncated mutants, but not ΔNΔSNARE (Fig. 6B), indicating that PRRSV nsp5 interacted with the N-terminal domain and SNARE domain of STX17.

## DISCUSSION

**FIG 7:**
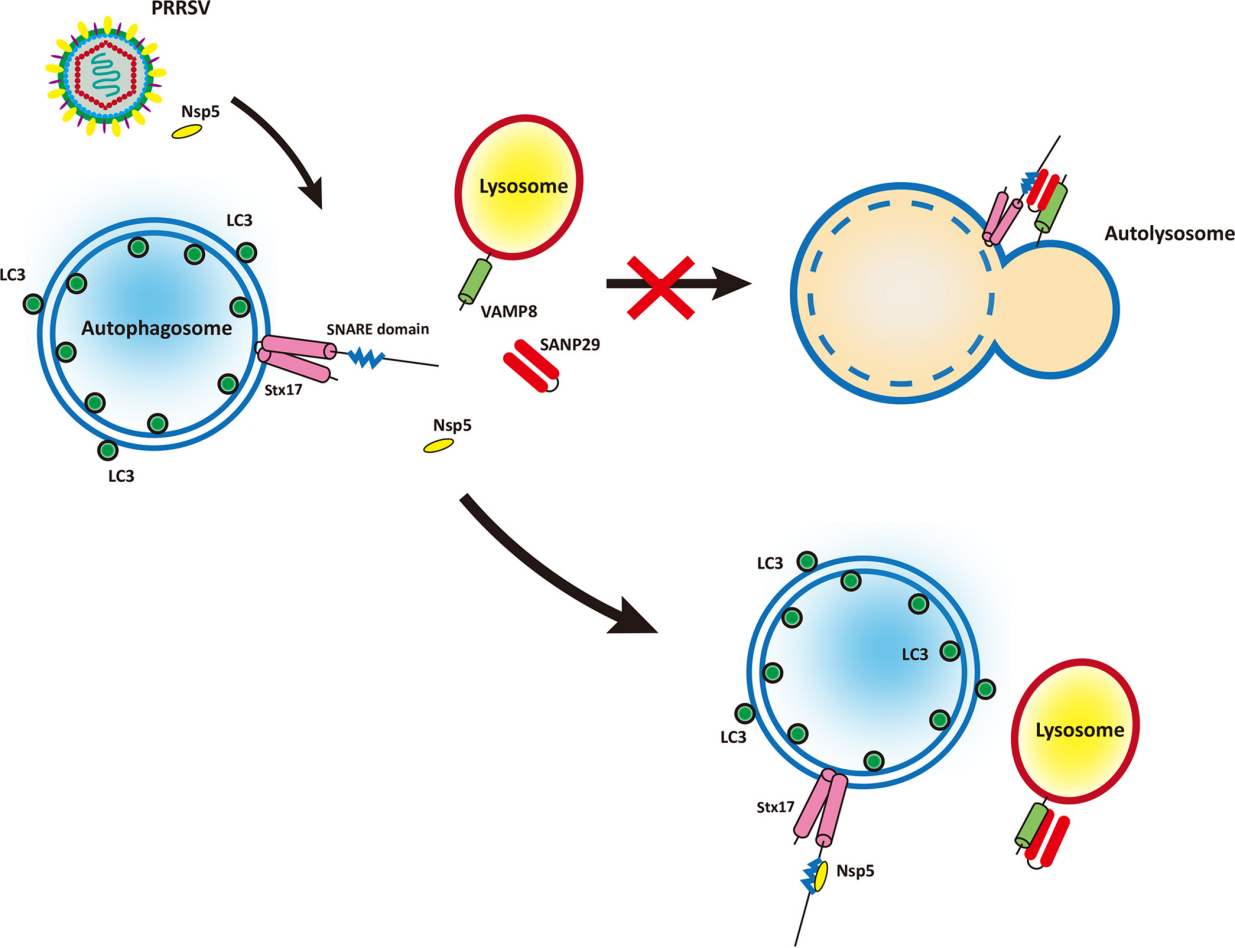
Model of PRRSV infection-induced and nsp5 overexpression-induced incomplete autophagy. The fusion of autophagosomes and lysosomes is predominantly mediated by the STX17-SNAP29-VAMP8 complex. Upon PRRSV infection or nsp5 overexpression, nsp5 interacts with STX17, thus preventing the interaction of STX17 and SNAP29, which subsequently impairs the assembly of the STX17-SNAP29-VAMP8 complex. Eventually, autophagosome-lysosome fusion is inhibited and autophagic flux is blocked.

Autophagy involves complex interactions with various cellular physiological processes, such as inflammation, interferon responses, antigen presentation, and apoptosis (40–44). Previous research has proved that autophagy is exploited by PRRSV to benefit its proliferation (23). Herein, we also demonstrated that CQ attenuated PRRSV proliferation in MARC-145 cells (Fig. S2). Therefore, illuminating the mechanism by which PRRSV regulates autophagy may provide an insight into PRRSV pathogenesis. In this study, we found that PRRSV infection, as well as nsp5 overexpression, induced the accumulation of autophagosomes by blocking their fusion with lysosomes. Further investigation revealed that nsp5 interacted with the N-terminal domain and SNARE domain of STX17 and disrupted the interaction between STX17 and SNAP29. This competitive interaction attenuated the assembly of the STX17-SNAP29-VAMP8 complex, which is pivotal to autophagosome-lysosome fusion (Fig. 7).

When complete autophagy occurs, autophagosomes accumulate and subsequently fuse with lysosomes to form autolysosomes before the degradation of the engulfed cytosolic cargos. However, various external stimuli, such as drugs (bafilomycin A1, chloroquine, and thapsigargin), can block autophagosome-lysosome fusion, which decreases the degradation of autophagosomes, resulting in autophagosome accumulation (45–49). This kind of impaired autophagy is called incomplete autophagy. Apart from drugs, several viruses have also been reported to induce incomplete autophagy through diverse viral proteins (50), such as matrix protein 2 of the influenza virus, nonstructural proteins 2C and 3C of coxsackievirus, helper protein Nef of the human immunodeficiency virus, and ORF3a of the severe acute respiratory syndrome coronavirus 2 (51–53). However, whether PRRSV infection induces complete or incomplete autophagy remains controversial. Wang et al. demonstrated that LAMP2 colocalizes with LC3 in PRRSV-infected cells, and CQ treatment further increased PRRSV-induced LC3-II levels, indicating that PRRSV infection induces complete autophagy (25). In contrast, Sun et al. reported that PRRSV infection induced incomplete autophagy because neither the colocalization of LAMP1 with LC3 nor the further accumulation of PRRSV-induced LC3-II in response to CQ treatment was observed (26). Here, we also found that PRRSV infection induced incomplete autophagy.

In terms of the viral proteins’ contribution to the PRRSV-mediated autophagy process, nsp2, nsp3, and nsp5 have been identified as inducers of autophagosome accumulation that act through various mechanisms. Nsp2 binds to 14-3-3ε via its tail domain, which plays an important role in nsp2-induced autophagy (26, 28); the cytoplasmic domain of nsp3 has been demonstrated to account for PRRSV nsp3-induced autophagy (27). Despite this, information on the mechanism by which nsp5 promotes autophagosome accumulation is still lacking. In this study, we found that PRRSV nsp5 failed to further increase LC3-II levels under CQ treatment, and tandem mCherry-EGFP-LC3 fluorescence protein exhibited as red-only puncta in cells expressing nsp5. These results illustrated that nsp5 attenuated the fusion of autophagosomes and lysosomes to induce incomplete autophagy, resulting in the accumulation of autophagosomes. In addition to nsp5, we found that PRRSV nsp2 and envelope (E) protein could upregulate the levels of LC3-II (Fig. S3A and B) and impair the autophagosome-lysosome fusion (Fig. S3C to F) as well. Besides, we also examined the effect of nsp6 of porcine enteric coronaviruses (porcine deltacoronavirus [PDCoV] and porcine epidemic diarrhea virus [PEDV]), which is an ortholog of PRRSV nsp5, on autophagic flux. As shown in Fig. S4A and B, nsp6 of both PDCoV and PEDV was able to further upregulate LC3-II levels in cells treated with CQ, which differed from the effect of PRRSV nsp5.

The STX17-SNAP29-VAMP8 complex mediates the fusion process between autophagosomes and lysosomes. Increasing evidence demonstrates that targeting SNARE proteins appears to be a common mechanism exploited by viruses to inhibit the fusion of autophagosomes and lysosomes. Hepatitis C virus prevents autolysosome formation by impairing STX17 expression (54). Similarly, coxsackievirus B3 (CVB3) represses the transcription and translation of STX17 to block autophagy flux and lysosomal function (55). Furthermore, proteases of CVB3, as well as those of enterovirus D68, also cleave SNAP29 to impair the formation of SNARE complexes and subsequent autophagosome-lysosome fusion (56). In addition, the P protein of human parainfluenza virus type 3 binds to SNAP29 and inhibits its interaction with STX17, thereby preventing autophagosome-lysosome fusion (16). Our results demonstrated that PRRSV nsp5 interacts with the N-terminal domain and SNARE domain of STX17 to antagonize the interaction of STX17 and SNAP29, resulting in the blockage of the STX17-SNAP29-VAMP8 complex assembly and subsequent autophagosome-lysosome fusion.

Because of the critical role of autophagy in protein degradation and the subsequent recycling of amino acids for the upkeep of protein synthesis, a growing number of studies have focused on the association between autophagy and translation. Dang et al. found that translation inhibitor-mediated translation shutoff promoted autophagosome biogenesis but blocked the formation of degradative autolysosomes by destroying functional lysosomes (57). Similar phenomena have also been observed during bacterial infections. Both α-hemolysin, a small pore-forming toxin of Staphylococcus aureus, and (S)-3-*oxo*-C_12_-homoserine lactone [(S)-3-*oxo*-C_12_-HSL], a quorum-sensing hormone of Pseudomonas aeruginosa, trigger α subunit of eukaryotic initiation factor 2 phosphorylation and subvert translation, which are required for autophagosome accumulation (58). In our previous study, we found that PRRSV infection, as well as nsp5 overexpression, induced host translation shutoff. However, whether the translation inhibition induced by PRRSV or nsp5 contributes to the occurrence of incomplete autophagy requires further investigation.

## MATERIALS AND METHODS

### Plasmids.

Expression constructs encoding nonstructural proteins of the PRRSV strain WUH3 (GenBank accession number HM853673) were amplified by PCR and cloned into pCAGGS vectors (59) to construct the following plasmids: pCAGGS-FLAG-nsp5, pCAGGS-FLAG-nsp56, pCAGGS-FLAG-nsp567, pCAGGS-FLAG-nsp67, pCAGGS-FLAG-nsp7, pCAGGS-HA-nsp5-GFP, pCAGGS-HA-nsp5, and pCAGGS-MYC-nsp5. The EGFP-tagged LC3 plasmid (pEGFP-LC3) was used as described previously (21). pmCherry-EGFP-LC3 (P0446), pLAMP1-mCherry (P10755), pDsRed2-Mito (P0142), and pLVX-TetOne-Puro (P1686) were purchased from MiaoLingBio. pDsRed-ER was obtained from BIOESN (BES20161ZT). cDNAs encoding SNAP29, VAMP8, and STX17 were amplified by PCR and cloned into pCAGGS vectors. pCMV-VSV-G and pCMV-gag-pol were gifted by Ping Qian at Huazhong Agricultural University, China.

### Antibodies and reagents.

A mouse monoclonal antibody against PRRSV nucleocapsid (N) protein was prepared in our lab (60). Mouse monoclonal anti-FLAG (MBL catalog number M185), anti-HA (MBL M180), and anti-MYC (MBL M047) antibodies, rabbit monoclonal anti-β-actin (ABclonal AC026) antibody, and rabbit polyclonal anti-GOSR1 (ABclonal A4316), anti-LC3 (Cell Signaling Technology clone D11), and anti-STX17 (ProteinTech 17815-1-AP) antibodies were used in our study. Chloroquine diphosphate (HY-17589) and rapamycin (AY-22989) were purchased from Med Chem Express. Earle's balanced salts solution (EBSS) was purchased from Sigma-Aldrich (E3024).

### Cells.

MARC-145 cells and HEK-293T cells were cultured in Dulbecco’s modified Eagle’s medium (Gibco 12100-061) supplemented with 10% fetal bovine serum (Seratech ST30-3302) and 50 U/mL penicillin-streptomycin (Thermo Fisher Scientific 15070063) in a 5% CO_2_ incubator.

HEK-293T cells stably expressing PRRSV nsp5 were generated as follows: the nsp5 sequence was cloned into a lentiviral gene expression vector (pLVX-TetOne-Puro) to construct the pLVX-TetOne-nsp5-Puro plasmid. Then, pLVX-TetOne-nsp5-Puro and helper plasmids (pCMV-VSV-G and pCMV-gag-pol) were cotransfected into HEK-293T cells. Forty-eight hours later, the supernatant was collected and centrifuged at 13,000 rpm for 3 h. The deposited pellet containing lentiviruses was resuspended and added to culture medium of fresh HEK-293T cells. Thirty-six hours later, the cells were digested and seeded into cell plates with 2 μg/mL puromycin. All surviving cells were amplified. To induce the expression of nsp5, 1 μg/mL doxycycline (Beyotime ST039A) was added to the cell medium for at least 36 h.

### Transient transfection.

For transfection into HEK-293T cells, the indicated doses of plasmids were mixed slowly with jetPRIME buffer and jetPRIME reagent (Polyplus 114-01) according to the manufacturer’s instructions. The mixture was incubated for 10 min at room temperature and then added to the culture medium of HEK-293T cells. After 4 h of incubation, the medium containing transfection complexes was removed and fresh medium was added.

For transfection into MARC-145 cells, Gibco Opti-MEM reduced serum medium (Thermo Fisher Scientific 31985070) was used to dilute indicated plasmids and P3000 reagent as well as Lipofectamine 3000 reagent (Thermo Fisher Scientific L3000008), respectively, according to the manufacturers’ instructions. After 5 min of incubation, the two mixtures were mixed together and, 30 min later, added to the culture medium of MARC-145 cells. After 4 h of incubation, the medium containing transfection complexes was removed and fresh medium was added.

### Indirect immunofluorescence assay.

Cells grown on coverslips were washed with phosphate-buffered saline (PBS) and fixed in 4% paraformaldehyde for 15 min at room temperature. Fixed cells were immediately permeabilized with precooled methanol for 10 min, blocked with 5% bovine serum albumin in PBS for 45 min, and incubated with the indicated antibodies for 1 h. After washing thoroughly, the cells were treated with DyLight 488-conjugated goat anti-mouse IgG (Abbkine A23210), DyLight 594-conjugated goat anti-rabbit IgG (Abbkine A23420), or DyLight 649-conjugated goat anti-mouse IgG (Abbkine A23610) for 1 h, followed by staining with 2-(4-amidinophenyl)-6-indolecarbamidine dihydrochloride (DAPI; Beyotime C1002) in PBS (1/2,000 dilution) for 15 min. Fluorescent images were acquired with an Olympus FV10 laser scanning confocal microscope.

### Coimmunoprecipitation assay.

Cells were washed two times with PBS and lysed at 4°C for 20 min in lysis buffer containing 50 mM Tris-HCl (pH 7.4), 150 mM NaCl, 1% NP-40, 0.5% deoxycholic acid, 0.1% SDS, and 1 mM EDTA. The proteins were immunoprecipitated overnight at 4°C with protein A+G agarose beads (Beyotime P2019) and affinity antibodies. The beads were washed three times with lysis buffer, and the immunoprecipitants were analyzed using immunoblotting.

### Western blot analysis.

Cells were washed three times with PBS and lysed in cell lysis buffer (Beyotime P0013). The cell lysate was mixed with 5× SDS loading buffer (Beyotime P0015), resolved with 12% acrylamide–SDS-PAGE buffer, and electroblotted onto polyvinylidene difluoride membranes. The membranes were blocked with 10% nonfat dry milk in Tris-buffered saline with 0.05% Tween 20 (TBST) for 1 h and incubated with the indicated antibodies at room temperature for 3 h. Following three washes with TBST, the membrane was incubated with horseradish peroxidase-conjugated goat anti-mouse IgG (Beyotime A0216) or goat anti-rabbit IgG (Beyotime A0208) at room temperature for 1 h. Signals were visualized using a ChemiDoc Touch imaging system from Bio-Rad and quantitated using ImageJ software.

### Luciferase reporter assay.

Cells cultured in 24-well plates were cotransfected with reporter plasmid (GRP78-*luc*, GRP94-*luc*, CHOP-*luc*, XBP-1-*luc*, UPRE-*luc*, and ERSE-*luc*) and pRL-TK plasmid (an internal control for normalization of the transfection efficiency) together with the indicated plasmids expressing GFP or GFP-nsp5. After 30 h, whole-cell lysates were prepared and firefly and *Renilla* luciferase activities were evaluated using a dual-luciferase reporter assay system (Promega) according to the manufacturer’s instructions. Data from three independently conducted experiments are shown as relative firefly luciferase activity normalized to *Renilla* luciferase activity.

### TCID_50_ assay.

Intracellular PRRSV was released by repeated freezing and thawing. Then, MARC-145 cells seeded in 96-well plates were infected with serial 10-fold dilutions of PRRSV samples in eight replicates for a 50% tissue culture infective dose (TCID_50_) assay. After incubation for about 96 h, the virus titers were calculated according to the cytopathic effect using the Reed-Muench method.
