# Supplementary material for: Porcine Reproductive and Respiratory Syndrome Virus nsp5 Induces Incomplete Autophagy by Impairing the Interaction of STX17 and SNAP29
Source: Microbiol Spectr. 2023 Feb 23;11(2):e04386-22. doi: 10.1128/spectrum.04386-22 (PMC10101144; doi:10.1128/spectrum.04386-22)
Supplement: Supplemental file 1 — Supplemental material. Download spectrum.04386-22-s0001.pdf, PDF file, 0.3 MB [file spectrum.04386-22-s0001.pdf]

Figure S1

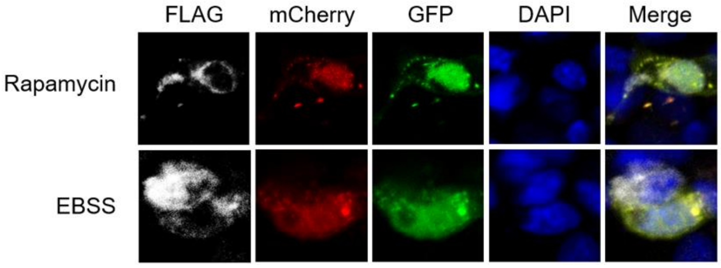

FIG. S1. Nsp5 blocks the autophagosome–lysosome fusion induced by EBSS or rapamycin

MARC-145 cells were co-transfected with pmCherry-EGFP-LC3 and pCAGGS-FLAG-nsp5, then treated with rapamycin or EBSS. Finally, cells were harvested for fluorescence analysis. Nuclei were stained with DAPI (blue).

Figure S2

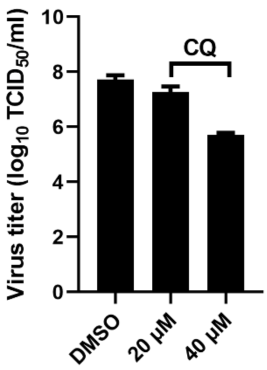

FIG. S2. CQ attenuates PRRSV proliferation

MARC-145 cells were infected with PRRSV (0.5 MOI) and treated with CQ (20 μM or 40 μM) for 36 h, then the cells were collected for $TCID_{50}$ assays.

Figure S3

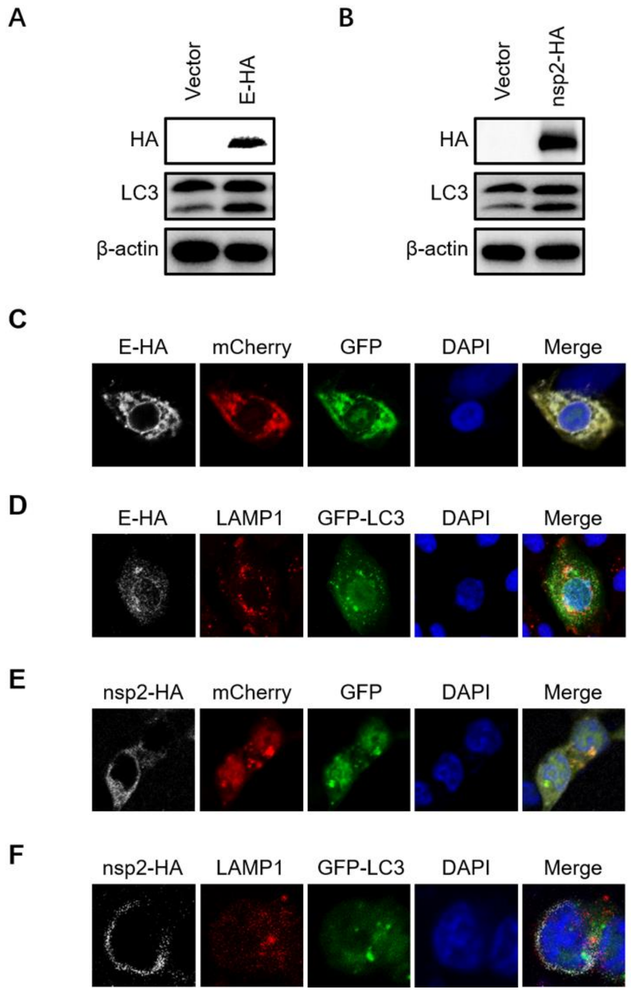

FIG. S3. PRRSV nsp2 and E protein induce incomplete autophagy

MARC-145 cells were transfected with HA-tagged plasmids encoding E protein (**A**) or nsp2 (**B**), respectively. After 30 h, the cells were harvested for western blot analysis to detect the expression levels of LC3. (**C** and **E**) MARC-145 cells were co-transfected with pmCherry-EGFP-LC3 and pCAGGS-HA-E (C) or pCAGGS-HA-nsp2 (E). Thirty hours later, cells were harvested for fluorescence analysis. (**D** and **F**) MARC-145 cells were co-transfected with pLAMP1-mCherry, pEGFP-LC3 and pCAGGS-HA-E (D) or pCAGGS-HA-nsp2 (F). Thirty hours later, cells were harvested for fluorescence analysis. Nuclei were stained with DAPI (blue).

Figure S4

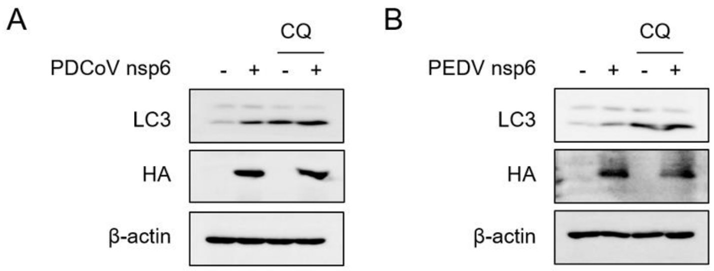

FIG. S4. PDCoV nsp6 and PEDV nsp6 induce complete autophagy

HEK-293T cells were transfected with plasmids expressing HA-tagged PDCoV nsp6 (**A**), PEDV nsp6 (**B**) or empty vector. After 24 h, cells were treated or mock-treated with CQ (20 μM) for another 12 h. Then cells were lysed for western blot analysis.
